# In Situ Synthesis, Crystallization Behavior and Mechanical Properties of Biodegradable Poly(Ethylene Succinate)/Talc Composites

**DOI:** 10.3390/polym18151812

**Published:** 2026-07-24

**Authors:** He Yang, Siyu Pan, Zhaobin Qiu

**Affiliations:** State Key Laboratory of Chemical Resource Engineering, Beijing University of Chemical Technology, Beijing 100029, China; 2025200363@buct.edu.cn

**Keywords:** poly(ethylene succinate), crystallization behavior, composites

## Abstract

Poly(ethylene succinate) (PES) is a promising biodegradable polyester with excellent thermal and mechanical properties; however, slow crystallization rate has seriously restricted its widespread application. PES/talc composites at low talc contents of 0.5 to 2.0 wt% were synthesized via an in situ polymerization method in this research. The chemical structure, thermal stability, crystallization behavior, crystalline morphology, crystal structure, and tensile mechanical properties of PES/talc composites were extensively investigated with various techniques and compared with those of neat PES. Talc remarkably enhanced the crystallization behavior of PES under different crystallization conditions as an effective nucleating agent. For instance, 2 wt% talc increased the melt crystallization temperature of PES from 46.1 to 58.9 °C at a cooling rate of 5 °C/min and shortened the crystallization half time to 1.95 min from 7.69 min at 66 °C. However, talc remained the crystallization mechanism and crystal structure of PES. The nucleation mechanism of talc on the crystallization of PES was further discussed. The exact nucleation mechanism was still uncertain, which needs further investigation. The mechanical properties of PES/talc composites were dependent on the talc content. For instance, 0.5 and 1 wt% talc decreased the tensile strength values to 34.1 ± 1.9 and 33.7 ± 2.1 MPa from 44.9 ± 1.3 MPa, while the elongation at break values increased to 675.3 ± 24.8% and 560.4 ± 11.9% from 508.0 ± 13.4%, respectively, indicating an increase in toughness. Although the mechanical properties of PES/talc2.0 (2 wt% talc) were inferior to those of neat PES and the other two composites, it still displayed relatively good mechanical properties.

## 1. Introduction

Poly(ethylene succinate) (PES) is one of the most important biodegradable aliphatic polyesters, displaying comparable mechanical properties to those of polypropylene and low-density polyethylene, favorable melt processability, and admirable biodegradability [1]. It shows a similar chemical structure to that of poly(butylene succinate) (PBS) with the only difference in the number of methylene groups in the repeating unit. Different to the successful industrialization of PBS by many companies in Japan, Thailand, and China, the industrialization of PES has rarely been achieved. PES can be synthesized from succinic acid (SA) and ethylene glycol (EG) through a two-stage melt polycondensation process, while PBS is synthesized in a similar method using SA and 1,4-butanediol (BDO) as the monomers. Compared to the relatively high cost of BDO, the price of EG is rather low. As a result, the cost of PES is more competitive than that of PBS. In addition, both SA and EG may also be derived from renewable resources; therefore, PES is also a fully biobased polyester if both the monomers used are from biomass. Even if one of the monomers is derived from biomass, PES can still be regarded as a biobased polyester. As a biodegradable and biobased aliphatic polyester, the synthesis, thermal and mechanical properties, and enzymatic degradation of PES were reported in the literature [2,3,4,5,6,7].

As a semicrystalline polyester, crystallization behavior study is of great interest and importance from both academic and practical application viewpoints. The crystallinity and morphology of PES not only affect the thermal and mechanical properties but also influence the thermal and enzymatic degradation behavior of PES. Similar to poly(L-lactic acid) (PLLA), the crystallization rate of PES is also very slow; therefore, PES may reach a fully amorphous state when a relatively fast cooling rate is used. Consequently, the crystallization behavior study of PES may be performed from different initial states, i.e., the cold crystallization from the amorphous state and the melt crystallization from the molten state. It should be emphasized that the most serious disadvantage of limiting the widespread end uses of PES is its slow crystallization rate. To address this problem, the proper utilization of nucleating agent is an effective way, which may supply additional nucleation sites, lower the nucleation activation energy, shorten the crystallization induction time, reduce the size and enhance the nucleation density of spherulites, and finally increase the overall crystallization rate of PES.

So far, many different nucleating agents have already been reported to accelerate the crystallization of PES, including clay, SiO_2_, WS_2_, graphene oxide (GO), thermal reduced graphene (TRG), carbon nanotubes (CNT), polyhedral oligomeric silsesquioxanes (POSS), cyanuric acid (CA), and cellulose nanocrystals (CNC) [8,9,10,11,12,13,14,15,16,17,18,19]. Among the above nucleating agents, CNC is a biobased and biodegradable nucleating agent for PES [16,17,18,19]. In previous studies, the influence of CNC on the nonisothermal melt crystallization, isothermal melt crystallization kinetics, and rheological behavior of PES/CNC composites were investigated in detail [17,18,19]. The small interfacial energy between CNC and PES favored the nucleation of PES on the surface of CNC [17]; as a result, CNC was an efficient nucleating agent for the crystallization of PES, demonstrating a relatively high nucleation efficiency value of 58.8% at only 0.5 wt% of CNC [18].

In addition to the above nucleating agents, the following nucleating agents have also recently been reported in the literature, such as tetramethylenedicarboxylic dibenzoylhydrazide (TMC306), octamethylenedicarboxylic dibenzoylhydrazide (TMC300), N,N-ethylenebis(12-hydroxystearamide) (EBH), layered metal phosphonate (PPZn), talc, boron nitride (BN), myo-inositol (MIS), dipentaerythritol (DPE), Xylitol (Xy), D-sorbitol (DS), and D-mannitol (DM) [20,21,22,23,24,25]. Yang et al. have recently investigated the influence of some novel nucleating agents, including TMC300, TMC306, EBH, and PPZn on the crystallization behavior of PES [20,21,22,23]. The nucleation mechanism was ascribed to the hydrogen bonding interaction between carbonyl group (of crystalline phase) and ester group (of amorphous phase) of PES with the nucleating agents [20,21,22,23]. Ye et al. have recently reported that two bio-derived hexahydric alcohols, i.e., MIS and DPE, remarkably increased the crystallization rate of PES through both hydrogen bonding interactions and epitaxial templating [24]. They further reported the effect of some sugar alcohols as effective nucleating agents for PES. They revealed that DM promoted the crystallization of PES via both intermolecular hydrogen bonding interactions and an epitaxial templating, while Xy and DS were primarily attributed to hydrogen bonding interactions [25].

Among the above-mentioned nucleating agents, talc is more competitive in cost than others. In the literature, talc was an efficient nucleating agent for some biodegradable polyesters, such as PLLA, poly(butylene adipate-*co*-terephthalate) (PBAT), and poly(3-hydroxybutyrate-*co*-3-hydroxyhexanoate) (P3HBHHx) [26,27,28]. However, to the best of our knowledge, the influence of talc on the crystallization behavior of PES has seldom been reported [23]. Yang et al. once prepared PES/talc composites via a solution and casting process. They found that the melt crystallization temperature of PES increased from 29.7 to 37.3 °C with an increase in talc content from 0.2 to 1 wt% at a cooling rate of 10 °C/min, while no crystallization exotherm was detected for neat PES [23]. Through an in situ polymerization method, PES/talc composites were prepared successfully in this work at low contents of talc from 0.5 to 2 wt%. The preparation of PES/talc composites via an in situ polymerization method should be more environmentally friendly than the solution and casting method in the literature [23], as no solvent was used in this process. The effect of talc on the crystallization behavior, spherulitic morphology, crystal structure, and mechanical properties of PES was further investigated in detail. The results revealed that talc remarkably promoted the crystallization behavior of PES under different conditions as an effective nucleating agent; moreover, talc content played a critical role of affecting the mechanical properties of PES. As a result, the wider practical application of PES may be extended in the presence of talc. This research is expected to be greatly interesting and important from both biodegradable polymer composite and polymer crystallization viewpoints.

## 2. Experimental Section

### 2.1. Materials

SA was purchased from Shandong Keyuan Biochemical Co., Ltd. (Heze, China). EG and tetrabutyl titanate (TBT) were provided by Tianjin Damao Chemical Reagent Factory (Tianjin, China). Talc (3000 mesh) was bought from Shanghai Macklin Biochemical Co., Ltd. (Shanghai, China). Without any further purification, all reagents were directly used.

### 2.2. In Situ Synthesis of PES/Talc Composites

**Scheme 1:**
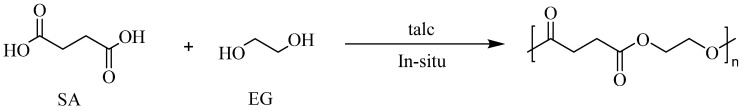
In situ synthesis of PES/talc composites.

Scheme 1 illustrates the in situ synthesis procedure of PES/talc composites. EG and talc were first charged into a three-neck flask and ultrasonicated for 1 h to obtain a uniformly dispersed mixture, followed by the addition of SA and 0.1 g/mL of TBT/toluene solution as a catalyst. In this research, the diacid and diol molar ratio was 1:1.1, while the mass of TBT is 0.5% of SA. In the esterification stage, the mixture was first heated in an oil bath to 170 °C under a nitrogen atmosphere with stirring and then gradually increased to 190 °C, and reacted for 3–4 h until no water was collected within 30 min. During the polycondensation step, the pressure was progressively lowered to below 200 Pa within 1 h. The temperature was then gradually raised to 220 °C, and the reaction lasted for 3–5 h till the rod-climbing phenomenon occurred. In a similar way, neat PES was also synthesized. The synthesized composites were abbreviated as PES/talc0.5, PES/talc1.0, and PES/talc2.0, with the number being the weight fraction of talc in the composite.

### 2.3. Characterizations

Hydrogen nuclear magnetic resonance (^1^H NMR) was performed at room temperature using deuterated chloroform (CDCl_3_) as a solvent on a Bruker AV 600 (Bruker, Billerica, MA, USA) to confirm the chemical structure of neat PES and PES/talc composites.

The chemical structures of neat PES and PES/talc composites were further verified by a Nicolet 6700 FT-IR spectrometer (Thermo Fisher Scientific, Madison, WI, USA) using the ATR technique with 32 scans and a resolution of 4 cm^−1^ from 4000 to 500 cm^−1^.

The intrinsic viscosity ([*η*]) values were measured by an Ubbelohde viscometer with a diameter of 0.4~0.5 mm at 25 °C for neat PES and PES/talc composites. All samples were dissolved in chloroform. [*η*] was calculated by the Solomon−Ciuta equation as follows [29].

(1) $$\eta =\frac{{\left[2\left(\frac{t}{t_{0}}-\ln\frac{t}{t_{0}}-1\right)\right]}^{\frac{1}{2}}}{c}$$

where *t*_0_ and *t* are the flow time of pure solvent and the solution, respectively, and *c* (0.4 g/dL in this research) is the concentration of the solution.

A scanning electron microscope (SEM, JEOL JSM-7800F, Tokyo, Japan) was used to observe the dispersion of talc in the PES matrix. Before observation, all samples were fractured into liquid nitrogen.

Thermogravimetric analysis (TGA) (TA Q50, TA Instrument, New Castle, DE, USA) was used to analyze the thermal stability of neat PES and PES/talc composites at a heating rate of 20 °C/min under a nitrogen (N_2_) atmosphere.

Differential scanning calorimetry (DSC) (TA Q100, TA Instrument, New Castle, DE, USA) was used to study the basic thermal property, nonisothermal melt crystallization behavior, and isothermal melt crystallization kinetics of neat and nucleated PES under a N_2_ atmosphere. In the case of the basic thermal property study, samples (about 4 mg) were heated to 150 °C at 40 °C /min, held for 3 min to erase thermal history, cooled to −50 °C at 60 °C/min, and reheated to 150 °C at 10 °C/min. In the case of the nonisothermal crystallization behavior study, samples were cooled to −50 °C at 5 °C/min from 150 °C after erasing previous thermal history. In the case of the isothermal crystallization kinetics study, samples were quenched at 60 °C/min to the crystallization temperature from the molten state and held for a sufficiently long time until complete crystallization.

The spherulitic morphologies were examined for neat and nucleated PES using a polarized optical microscope (POM) (Olympus BX51, Tokyo, Japan) with a hot stage (THMS 600, Linkam, Redhill, UK).

Wide angle X-ray diffraction (WAXD) profiles of neat PES and PES/talc composites were obtained on a Rigaku Ultima X-ray diffractometer (Tokyo, Japan) (*λ* = 0.154 nm, 40 kV, and 40 mA) at a scanning rate of 5°/min over the range 2*θ* from 5 to 50°. Samples were first crystallized in an oven at 64 °C for 3 h from the molten state.

The tensile mechanical properties were measured at room temperature on a K-TEST KXWW-20C electronic universal testing machine with a crosshead speed of 20 mm/min. The GB/T 1040.3-2006 standard was used in this research. Dumbbell specimens (thickness: 0.5 mm, width: 4 mm, length: 50 mm, gauge length: 18 mm) were prepared by melt pressing samples into films using a hot press machine at 125 °C under a pressure of 7 MPa for 5 min and then quickly quenched into ice water. At least 3 specimens were used for each test to obtain the average values.

## 3. Results and Discussion

### 3.1. ^1^H NMR and FTIR Spectra Studies

**Figure 1:**
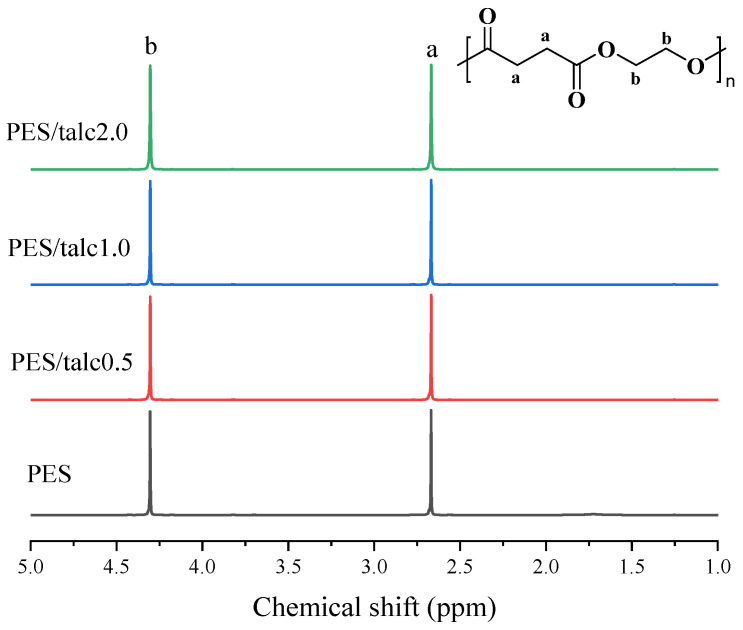
^1^H NMR spectra for both neat PES and PES/talc composites.

Figure 1 illustrates the ^1^H NMR spectra for both neat PES and PES/talc composites. PES/talc composites displayed the similar ^1^H NMR spectra to that of neat PES. Two signals clearly appeared for neat PES and PES/talc composites. One signal at 4.30 ppm was from the protons of EG, while the other at 2.67 ppm was from the protons of SA. The ^1^H NMR spectra obviously indicated the successful synthesis of neat PES and PES/talc composites through the two-step esterification and condensation reaction.

**Figure 2:**
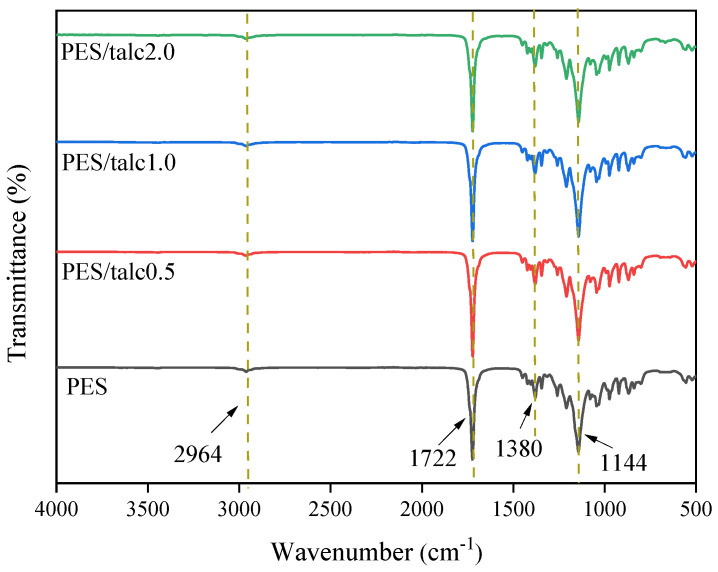
FTIR spectra of neat PES and PES/talc composites.

The chemical structures were further investigated with FTIR. As displayed in Figure 2, the main characteristic peaks of neat and nucleated PES were described as follows: C=O stretching (around 1722 cm^−1^), C-O-C stretching (around 1144 cm^−1^), C-H stretching (around 2964 cm^−1^), and C-H bending (around 1380 cm^−1^) [20,21,22,23,24,25]. In addition, the presence of talc did not significantly affect the location and intensity of the above main characteristic peaks, indicating that the interaction between PES and talc was slight and could be neglected. In brief, both ^1^H NMR and FTIR spectra confirmed the successful synthesis of neat PES and PES/talc composites.

The *η* values were measured with an Ubbelohde viscometer at 25 °C and calculated through the Solomon−Ciuta equation [29]. The *η* value of neat PES was 0.54 dL/g, while those of PES/talc composites were around 0.52 to 0.61 dL/g. The *η* values were 0.55, 0.61, and 0.52 dL/g, respectively, with the talc content increasing from 0.5 to 2.0 wt%. The small difference in the *η* values may be related to the following two facts. On one hand, the detailed polycondensation time for each sample was slightly different. On the other hand, the presence of talc may hinder the molecular weight increase of PES, especially at 2 wt%. As the Mark–Houwink constants are not available for PES in chloroform, the viscosity-average molecular weight could not be further derived from the above *η* values. As neat PES and PES/talc composites are not soluble in tetrahydrofuran (THF), the *M*_w_ and *M*_n_ values could not be easily measured with Gel Permeation Chromatography (GPC) using THF as the solvent. However, Papageorgiou et al. once reported that the weight-average molecular weight (*M*_w_) and number-average molecular weight (*M*_n_) were about 43,700 and 17,100 g/mol for a PES sample with the *η* value of 0.57 dL/g using chloroform as a solvent in the literature [30]. Because the *η* values of neat PES and PES/talc composites in this research were close to 0.57 dL/g, neat PES and PES/talc composites may show similar and relatively high molecular weights to that reported by Papageorgiou et al. [30]. As the molecular weight was an important factor of influencing the crystallization behavior, mechanical properties, and even biodegradation behavior, we tried our best to diminish this influence by synthesizing the samples with a narrow range of *η* values between 0.52 and 0.61 dL/g in this research. Although the influence could not be excluded completely, it should not be the key factor of influencing crystallization behavior and mechanical properties.

**Figure 3:**
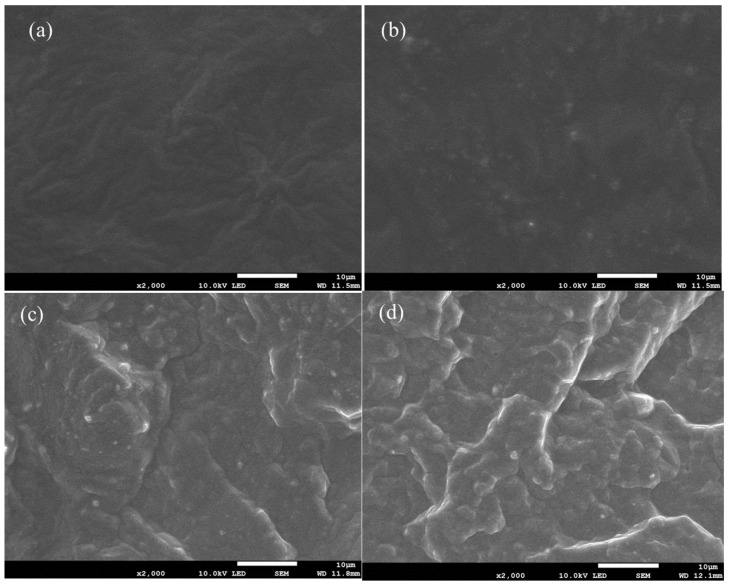
SEM images showing the fracture surfaces of (**a**) neat PES, (**b**) PES/talc0.5, (**c**) PES/talc1.0, and (**d**) PES/talc 2.0.

The dispersion of talc in PES matrix was of great importance to discuss the effect of talc on the crystallization behavior and mechanical properties of PES. Therefore, SEM was used to observe the dispersion of talc in the PES matrix. Figure 3 displays the SEM images of the fracture surfaces of neat PES and PES/talc composites. As shown in Figure 3a, PES displayed a smooth surface, while in the case of PES/talc composites, talc was observed. When the talc content was 1 wt% and below, talc evenly dispersed in the PES matrix. When the talc content was 2 wt%, some aggregation of talc was observed.

### 3.2. Thermal Stability and Basic Thermal Property Studies

**Figure 4:**
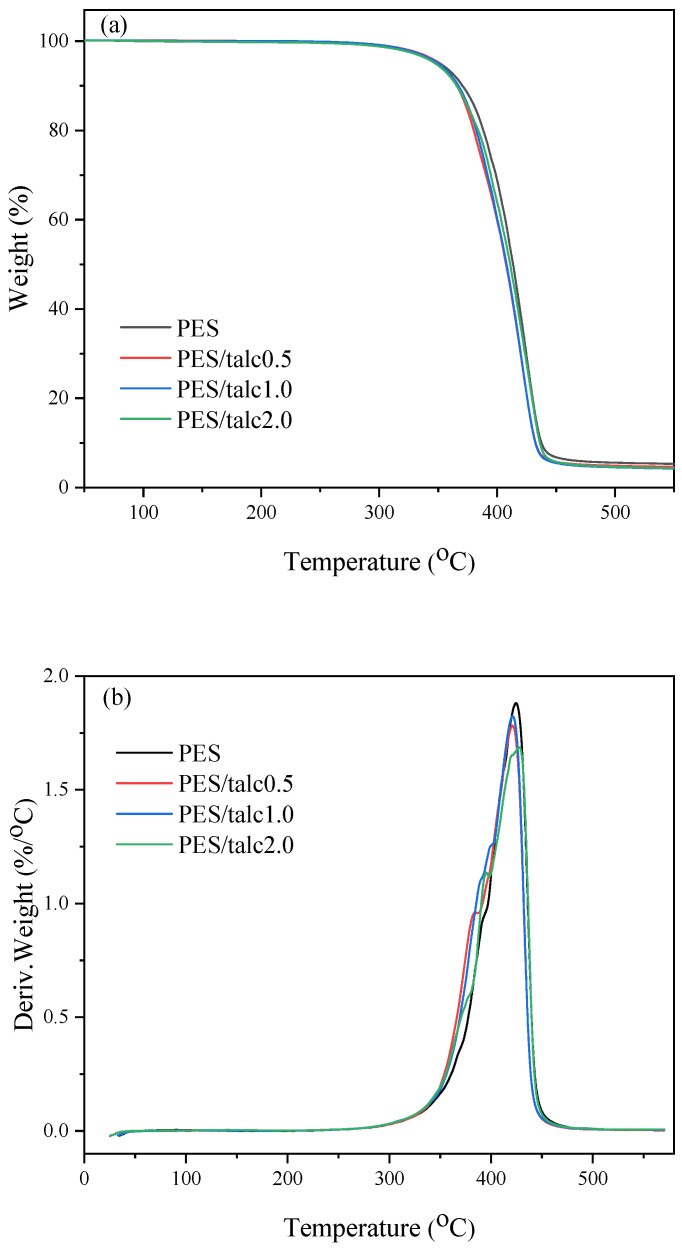
(**a**) TGA and (**b**) DTG results for neat PES and PES/talc composites.

The thermal stability was measured with TGA at 20 °C/min for all samples. As clearly depicted in Figure 4, one-step thermal degradation process was observed for both neat PES and PES/talc composites. From Figure 4a, the degradation temperature at 5 wt% weight loss (*T*_d_) value was determined to be 351.6 °C for neat PES, while they varied slightly between 348.1 and 351.1 °C for the PES/talc composites. Figure 4b displays the differential TG (DTG) results, from which the temperature at the maximum decomposition rate (*T*_max_) values for neat PES and PES/talc composites were measured. The *T*_max_ values varied between 420.9 and 427.8 °C. The small difference in the *T*_max_ values may be related to the difference in the molecular weights. The above results revealed that the presence of low contents of talc did not influence significantly the thermal stability of PES. The in situ synthesized PES/talc composites still maintained high thermal stability, demonstrating the *T*_d_ values of around 350 °C, which must be greatly important from a polymer melt processing viewpoint.

**Figure 5:**
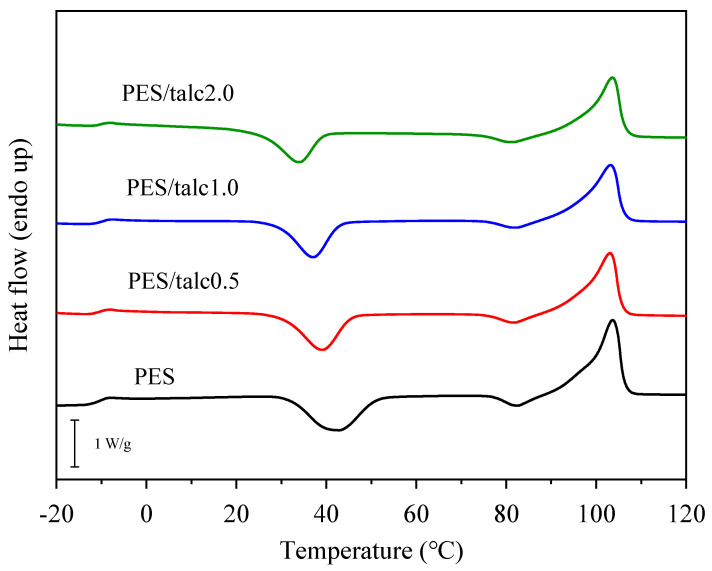
DSC heating traces of neat PES and PES/talc composites at 10 °C/min after cooling from the molten state at 60 °C/min.

The basic thermal property study was further performed with DSC. Figure 5 depicts the DSC heating traces at 10 °C/min for both neat PES and PES/talc composites after quenching from the molten state at 60 °C/min. During the fast cooling process at 60 °C/min, neat PES and PES/talc0.5 did not crystallize, while PES/talc1.0 and PES/talc2.0 showed crystallization exotherms, indicating that talc promoted the melt crystallization at a fast cooling process at a talc content of 1 wt% and above. Figure S1 in the Supplementary Material displays the above results. As illustrated in Figure 5, talc showed a slight influence on the glass transition temperature (*T*_g_) and melting temperature (*T*_m_) of PES. The *T*_g_ varied slightly between −10.3 and −9.6 °C; meanwhile, the *T*_m_ values remained almost unchanged at around 103 °C for all samples. The low content of talc decreased the cold crystallization temperature (*T*_ch_) to 33.9 °C for PES/talc2.0 from 42.7 °C for neat PES, suggesting that talc apparently promoted the cold crystallization of PES. Table 1 lists the relevant basic thermal properties, including the *T*_g_, *T*_m_, *T*_ch_, cold crystallization enthalpy (Δ*H*_ch_), and melting enthalpy (Δ*H*_m_).

### 3.3. Crystallization Behavior and Crystalline Morphology Studies

**Figure 6:**
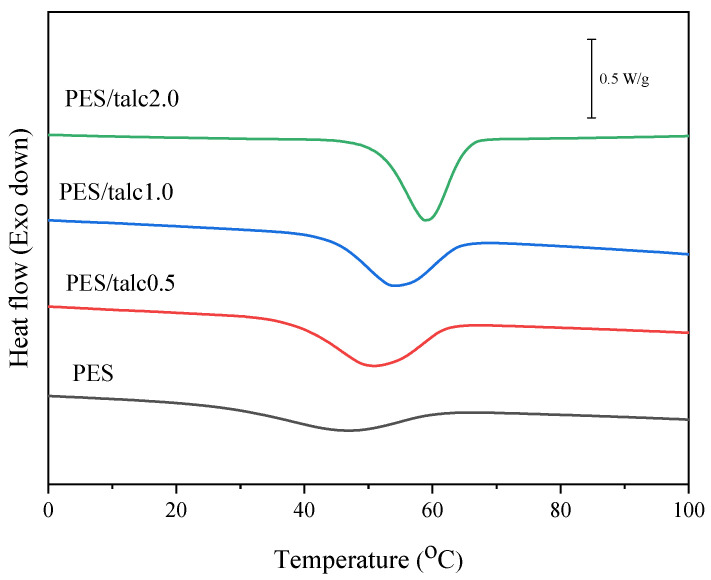
DSC cooling traces of neat PES and PES/talc composites at 5 °C/min.

The melt crystallization behavior at 5 °C/min was further studied with DSC from the molten state. As depicted in Figure 6, neat PES displayed a melt crystallization temperature (*T*_cc_) of 46.1 °C with a melt crystallization enthalpy (Δ*H*_cc_) of 30.7 J/g. PES/talc composites displayed obviously greater *T*_cc_ and Δ*H*_cc_ values than neat PES, indicating that talc enhanced the melt crystallization behavior of PES as an efficient heterogeneous nucleating agent. In addition, the enhancement effect became more pronounced with an increase in talc content. For instance, PES/talc2.0 displayed a *T*_cc_ of 58.9 °C with a Δ*H*_cc_ of 57.6 J/g.

**Figure 7:**
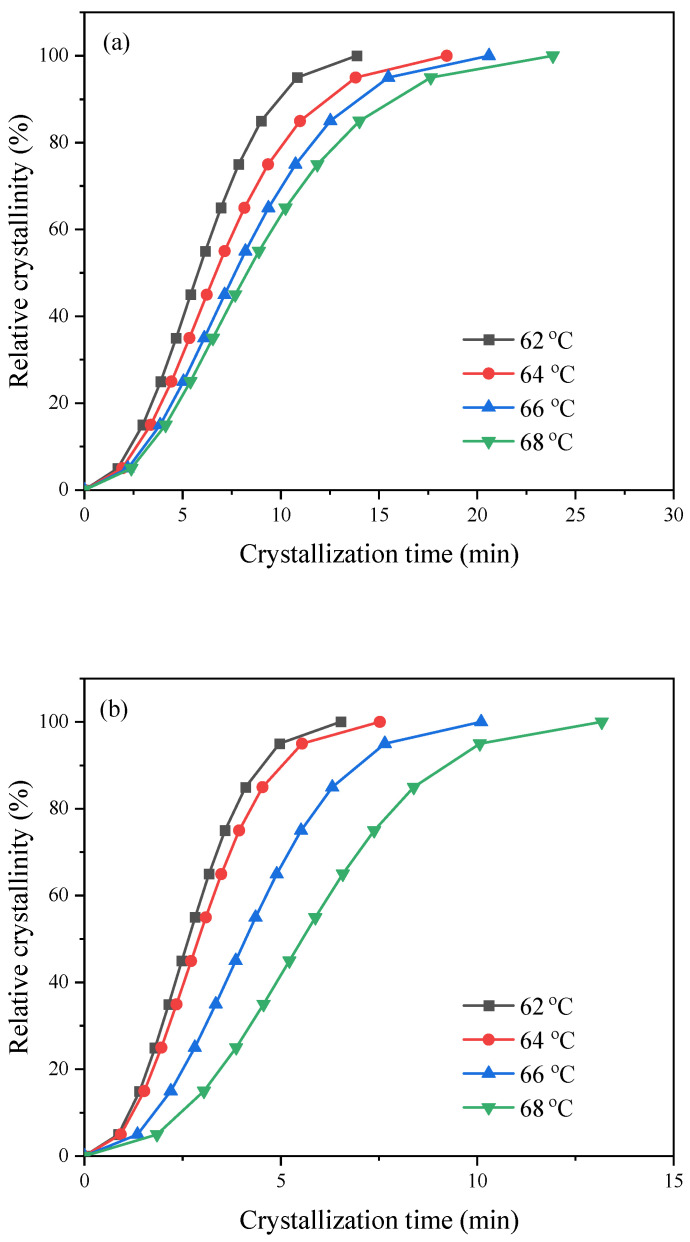
Development of relative crystallinity with crystallization time for (**a**) neat PES and (**b**) PES/talc1.0.

We further studied the isothermal melt crystallization kinetics in this section. Figure 7 depicts the plots of relative crystallinity versus crystallization time for neat PES and PES/talc1.0 as an example. The relevant results for PES/talc0.5 and PES/talc2.0 are displayed in Figure S2 in the Supplementary Material. With increasing crystallization temperature (*T*_c_), crystallization time became gradually longer for both neat and nucleated PES due to the reduced degree of supercooling, the driving force for polymer crystallization. In addition, crystallization time became significantly shorter with an increase in talc content at the same *T*_c_, confirming again the nucleation agent effect of talc.

**Figure 8:**
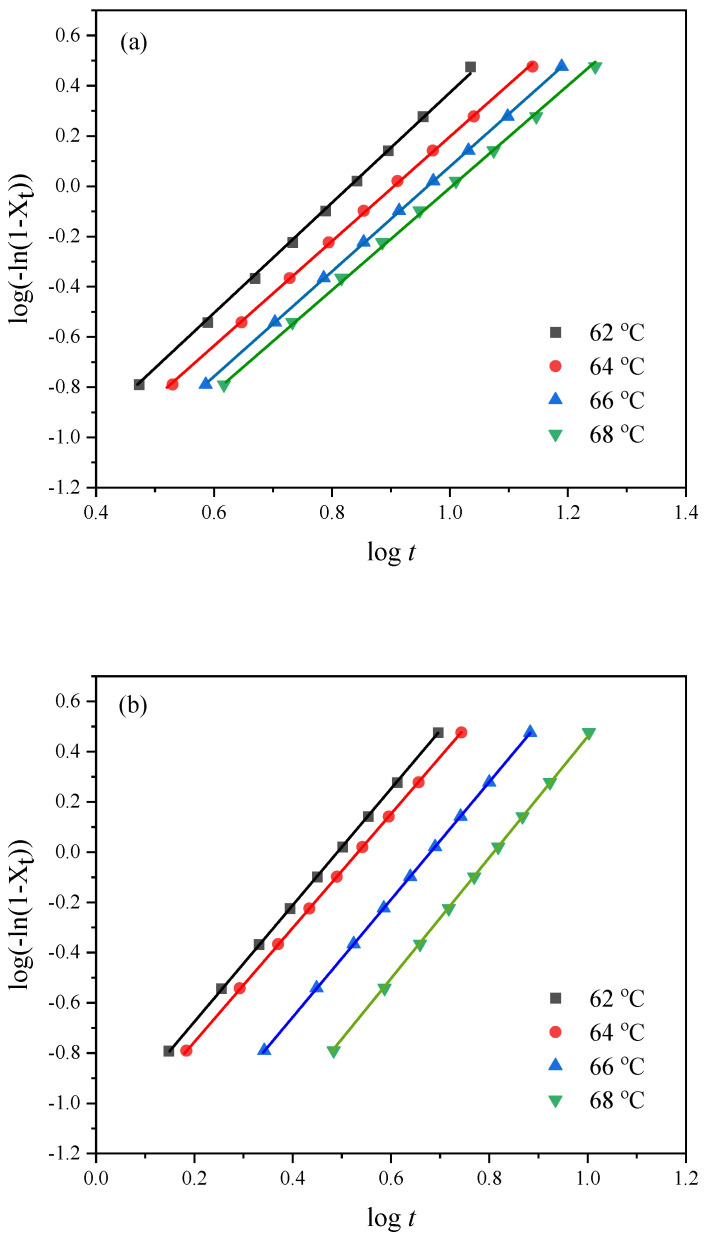
Avrami plots of (**a**) neat PES and (**b**) PES/talc1.0.

The classical Avrami equation was further used to fit the isothermal melt crystallization kinetics of neat PES and PES/talc composites [31,32]. Relative crystallinity (*X*_t_) develops with crystallization time (*t*) as follows:

(2) $$1-\;X_{t}=\exp\;(-kt^{n})$$

where *n* is the Avrami exponent, reflecting the crystallization mechanism, while *k* is the overall crystallization rate, involving both nucleation and growth processes [31,32]. The Avrami plots are depicted in Figure 7 for neat PES and PES/talc1.0 as an example. Figure S3 in the Supplementary Material shows the results for the other two samples. Figure 8 shows four almost parallel straight fitting lines, suggesting that the Avrami equation may fit the crystallization process. Table 2 lists the related Avrami parameters. From Table 2, the *n* values varied slightly between 2.0 and 2.5, despite the talc content within the investigated *T*_c_, suggesting that talc remained the crystallization mechanism of PES [33]. The following morphology study supported that PES crystallized through a spherulitic growth crystallization with heterogeneous nucleation mechanism. According to this mechanism, the *n* value should be an integer of 3; however, the experimentally obtained *n* values are usually smaller than 3 due to many different reasons, such as geometric, kinetic, or experimental reasons, which thus tends to reduce the apparent *n* values. In addition, the *k* values increased with a decrease in *T*_c_ for both neat and nucleated PES, indicating a faster crystallization rate at lower *T*_c_; moreover, at the same *T*_c_, the *k* values increased with increasing talc content, suggesting talc accelerated the isothermal crystallization of PES. For a direct comparison of crystallization rate of neat and nucleated PES at different *T*_c_ values, crystallization half time (*t*_1/2_), calculated through the following equation and listed in Table 2, was utilized in this research:

(3) $$t_{1/2}={\left(\frac{\ln2}{k}\right)}^{1/n}$$

For both neat and nucleated PES, the *t*_1/2_ values increased with an increase in *T*_c_, indicating a slower crystallization rate at a higher *T*_c_ due to the decreased degree of supercooling. In addition, the *t*_1/2_ values increased with a decrease in talc content, indicating a slower crystallization rate at smaller talc content.

**Figure 9:**
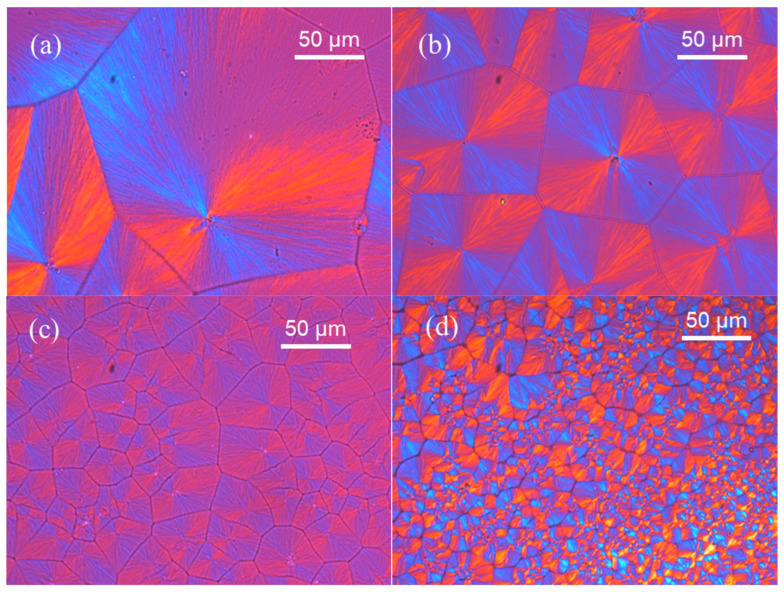
Spherulitic morphology of (**a**) neat PES, (**b**) PES/talc0.5, (**c**) PES/talc1.0, and (**d**) PES/talc2.0.

Figure 9 illustrates the POM images, displaying PES spherulites with different numbers and sizes after crystallizing at 66 °C. From Figure 9, increasing the talc content obviously increased the number and decreased the size of PES spherulites, indicating that talc was an efficient nucleating agent. The presence of talc provided more additional sites for the nucleation of PES; as a result, the nucleation density of PES spherulites increased accordingly. The plausible nucleation mechanism of talc on PES will be discussed in the following section.

### 3.4. Crystal Structure and Nucleation Mechanism Studies

**Figure 10:**
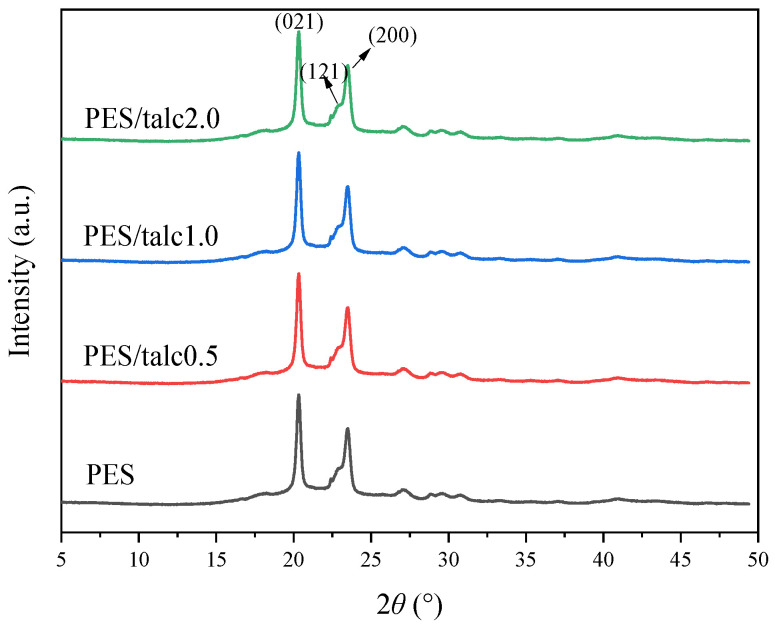
WAXD results of neat PES and PES/talc composites.

Figure 10 displays the WAXD profiles after neat PES and PES/talc composites first crystallized at 64 °C for 3 h. Both neat and nucleated PES showed three strong diffraction peaks at 2*θ* of 20.3°, 22.9°, and 23.5°, corresponding to (021), (121), and (200) crystal planes of PES, respectively [34]. The above result indicated that the crystal structure of PES remained unchanged despite the presence of talc. In addition, the crystallinity values were calculated from Figure 9 using a Jade software, which increased slightly from about 45 ± 3% for neat PES to 50 ± 3% for the composites.

From the above studies, talc promoted the crystallization of PES as an efficient nucleating agent under different conditions; therefore, it was necessary to discuss the possible nucleation mechanism. In polymer crystallization, many different kinds of nucleation mechanisms have been proposed. Among them, the following two kinds of nucleation mechanism are widely accepted. One is the chemical nucleation, and the other is the epitaxial nucleation [35]. In the literature, Yang et al. and Ye et al. also discussed the possible nucleation mechanism of PES with some nucleating agents [20,21,22,23,24,25]. They mainly compared the lattice parameters between PES and nucleating agents; moreover, they also used FTIR to detect the intermolecular interactions between PES and nucleating agents. From these results, they concluded an epitaxial nucleation mechanism in their studies [20,21,22,23,24,25]. In the case of PES/talc composites, the chemical nucleation was impossible on the basis of their chemical structures. Therefore, it should be necessary to compare the crystal structures of PES and talc. Ueda et al. first reported an orthorhombic unit cell with lattice parameters of *a* = 0.760 nm, *b* = 1.075 nm, and *c* = 0.833 nm for the crystal structure of PES [34]. Rayner et al. proposed that talc displayed a triclinic unit cell with lattice parameters of *a* = 0.529 nm, *b* = 0.918 nm, *c* = 0.950 nm, *α* = 90.57°, *β* = 98.91°, and *γ* = 90.03° [36]. After careful comparison of the crystal structures of PES and talc, the most likely epitaxial mechanism is that the (100) or (010) crystal plane of PES was oriented parallel to the (001) basal surface of talc, with the PES chain axis [001] (0.833 nm) aligned along the talc [010] direction (0.918 nm). The resulting lattice mismatch was merely 9.3%, falling within the generally accepted criterion for epitaxial nucleation. As a result, the possible nucleation mechanism in PES/talc composites may be attributed to the epitaxial nucleation mechanism of PES induced by talc. It should be emphasized that the above epitaxial nucleation mechanism was only plausible when experimental evidence were further obtained, such as electron diffraction, Transmission Electron Microscope (TEM), oriented-growth POM decoration experiments, or grazing-incidence WAXD. In addition to the above plausible epitaxial nucleation mechanism, another possible nucleation mechanism is the heterogeneous nucleation, which more commonly proceeds by simple surface energy reduction/geometric considerations without crystallographic epitaxy. In brief, the exact nucleation mechanism is still uncertain in this research, which needs further investigation.

### 3.5. Tensile Mechanical Property Study

**Figure 11:**
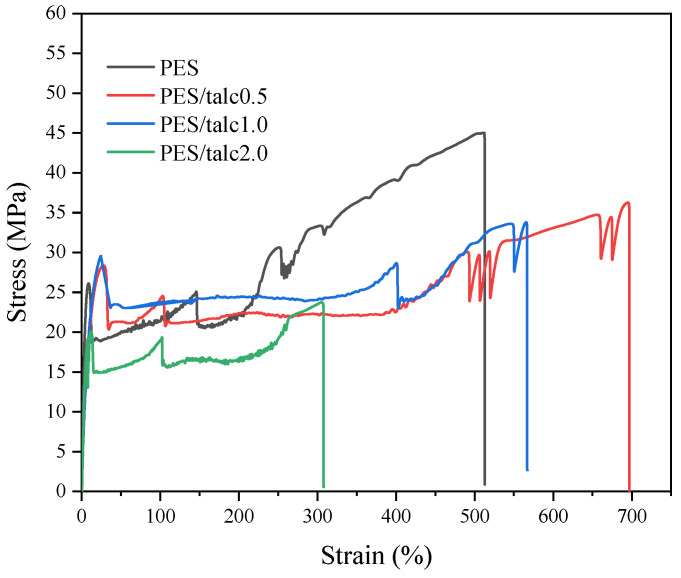
Stress–strain curves of neat PES and PES/talc composites.

Figure 11 illustrates the typical stress–strain curves of semicrystalline polymers for both neat PES and PES/talc composites, displaying yielding at low strain and fracture at high strain. Both neat PES and PES/talc composites experienced a typical deformation behavior as follows, i.e., first an initial elastic deformation, then stress yielding, neck propagation, strain hardening, and final fracture. In addition, some zigzag curves were also found, which was attributed to the occurrence of stress oscillation [37]. A similar result was also reported in other semicrystalline biodegradable polyester, such as PBS [37]. Table 3 lists the detailed mechanical properties’ data. Neat PES shows a Young’s modulus (*E*_t_) of 608.7 ± 7.1 MPa, a tensile strength (*σ*_b_) of 44.9 ± 1.3 MPa, and an elongation at break (*ε*_b_) of 508.0 ± 13.4%. The mechanical properties of PES/talc composites were dependent on the talc content. When the talc content was 1 wt% and below, PES/talc composites still displayed excellent mechanical properties. Compared with those of neat PES, the composites showed decreased *σ*_b_ values but increased *ε*_b_ values, indicating an increase in toughness. For instance, 0.5 and 1 wt% talc decreased the *σ*_b_ values to 34.1 ± 1.9 and 33.7 ± 2.1 MPa, while the *ε*_b_ values increased to 675.3 ± 24.8% and 560.4 ± 11.9%, respectively. When the talc content was 2 wt%, the mechanical properties of the composite were inferior to those of neat PES and the other two composites, because the relatively high content of talc acted as the defects and weakened the mechanical properties of PES. However, it should be emphasized that even PES/talc2.0 still displayed relatively good mechanical properties in this research.

It should also be noted that the biodegradation behavior of PES/talc composites may be different from that of neat PES, because talc not only changed the crystallinity but also changed the spherulitic morphology of PES, which would in turn influence the biodegradation behavior. Such a study should be performed carefully and would be reported in the forthcoming research.

## 4. Conclusions

In this research, the in situ synthesis, crystallization behavior, and mechanical properties of biodegradable PES/talc composites were systematically studied with ^1^H NMR, FTIR, TGA, DSC, POM, WAXD, and a tensile test. Through an in situ polymerization method, PES/talc composites with [*η*]) values ranging from 0.52 to 0.61 dL/g were synthesized at low talc contents of 0.5 to 2.0 wt%. The chemical structures of neat PES and PES/talc composites were confirmed through both the ^1^H NMR and FTIR studies. The thermal stability, basic thermal parameters, crystallization behavior, crystal structure, and tensile mechanical properties of PES/talc composites were extensively investigated and compared with those of neat PES. Low contents of talc showed a slight influence on the *T*_g_ and *T*_m_ of PES; however, as an efficient nucleating agent, talc significantly promoted the crystallization behavior of PES under different conditions. In the case of the cold crystallization behavior study at a heating rate of 10 °C/min, the *T*_ch_ values decreased from 42.7 °C for neat PES to 33.9 °C for PES/talc2.0. In the case of the melt crystallization behavior study at a cooling rate of 5 °C/min, the *T*_cc_ values remarkably increased from 46.1 °C for neat PES to 58.9 °C for PES/talc2.0; moreover, the Δ*H*_cc_ values also obviously increased to 57.6 J/g for PES/talc2.0 from 30.7 J/g for neat PES. For the isothermal melt crystallization kinetics study, talc apparently increased the overall crystallization rate and still maintained the crystallization mechanism of PES. For instance, the *t*_1/2_ values remarkably decreased to 1.95 min for PES/talc2.0 from 7.69 min for neat PES at the same *T*_c_ of 66 °C. The crystalline morphology study revealed an obvious decrease in the size and an increase in the nucleation density of PES spherulites, directly evidencing the nucleating agent role of talc. PES/talc composites displayed the same crystal structure as neat PES; moreover, talc slightly increased the crystallinity values in the composites. We further discussed the plausible nucleation mechanism. However, the exact nucleation mechanism is still uncertain at present, which needs further investigation. Finally, the mechanical properties of neat PES and PES/talc composites were studied, which showed a talc content dependence. When the talc content was 1 w% and below, compared with those of neat PES, the composites showed decreased *σ*_b_ values but increased *ε*_b_ values, indicating an increase in toughness. For instance, 0.5 and 1 wt% talc decreased the *σ*_b_ values to 34.1 ± 1.9 and 33.7 ± 2.1 MPa, while the *ε*_b_ values increased to 675.3 ± 24.8% and 560.4 ± 11.9%, respectively. However, the mechanical properties of the composite were inferior to those of neat PES and the other two composites, when the talc content was 2 wt%.

## Figures and Tables

**Table 1:** Summary of basic thermal parameters for neat PES and PES/talc composites.

Samples	*T*_g_ (°C)	*T*_ch_ (°C)	Δ*H*_ch_ (J/g)	*T*_m_ (°C)	Δ*H*_m_ (J/g)	*T*_cc_ (°C)	Δ*H*_cc_ (J/g)	*T*_d_ (°C)
PES	−10.3	42.7	51.0	103.7	64.9	46.1	30.7	351.6
PES/talc0.5	−10.1	39.1	44.5	103.1	60.9	50.7	48.8	351.1
PES/talc1.0	−9.6	37.0	40.9	103.2	60.8	54.0	47.0	350.6
PES/talc2.0	−10.1	33.9	38.4	103.6	61.3	58.9	57.6	348.1

**Table 2:** Isothermal crystallization kinetics data of neat PES and PES/talc composites.

Samples	*T*_c_ (°C)	*n*	*k* (min^−n^)	*t*_1/2_ (min)
PES	62	2.2	1.51 × 10^−2^	5.72
	64	2.1	1.30 × 10^−2^	6.74
	66	2.1	9.74 × 10^−3^	7.69
	68	2.0	9.07 × 10^−3^	8.41
PES/talc0.5	62	2.2	5.41 × 10^−2^	3.13
	64	2.2	3.11 × 10^−2^	4.03
	66	2.3	1.70 × 10^−2^	5.15
	68	2.4	6.71 × 10^−3^	6.65
PES/talc1.0	62	2.3	7.25 × 10^−2^	2.65
	64	2.3	6.17 × 10^−2^	2.90
	66	2.3	2.55 × 10^−2^	4.10
	68	2.4	1.13 × 10^−2^	5.53
PES/talc2.0	66	2.3	1.46 × 10^−1^	1.95
	68	2.5	7.10 × 10^−2^	2.46
	70	2.3	4.76 × 10^−2^	3.13
	72	2.5	1.60 × 10^−2^	4.42

**Table 3:** Summary of tensile mechanical properties’ data.

Samples	*E*_t_ (MPa)	*σ*_b_ (MPa)	*ε*_b_ (%)
PES	608.7 ± 7.1	44.9 ± 1.3	508.0 ± 13.4
PES/talc0.5	578.5 ± 19.7	34.1 ± 1.9	675.3 ± 24.8
PES/talc1.0	627.2 ± 9.8	33.7 ± 2.1	560.4 ± 11.9
PES/talc2.0	413.1 ± 17.1	23.7 ± 0.8	325.5 ± 8.3

## Data Availability

Data are available upon reasonable request from the authors.

## Supplementary Materials

The following supporting information can be downloaded at: https://www.mdpi.com/article/10.3390/polym18151812/s1, Figure S1: DSC cooling traces of neat PES and PES/talc composites at 60 °C/min; Figure S2: Plots of relative crystallinity versus crystallization time for (a) PES/talc0.5 and (b) PES/talc2.0; Figure S3: Avrami plots of (a) PES/talc0.5 and (b) PES/talc2.0.
