# Supplementary material for: In Situ Synthesis, Crystallization Behavior and Mechanical Properties of Biodegradable Poly(Ethylene Succinate)/Talc Composites
Source: Polymers (Basel). 2026 Jul 24;18(15):1812. doi: 10.3390/polym18151812 (PMC13468690; doi:10.3390/polym18151812)
Supplement: Supplementary file 1 [file polymers-18-01812-s001.zip › polymers-4443667-supplementary.pdf]

In situ Synthesis, Crystallization Behavior and Mechanical Properties of Biodegradable Poly(ethylene succinate)/Talc Composites

He Yang, Siyu Pan*, Zhaobin Qiu*

State Key Laboratory of Chemical Resource Engineering, Beijing University of Chemical Technology, Beijing 100029, China

Email:2022400087@buct.edu.cn (S Pan)

E-mail: qiuzb@mail.buct.edu.cn (Z Qiu)

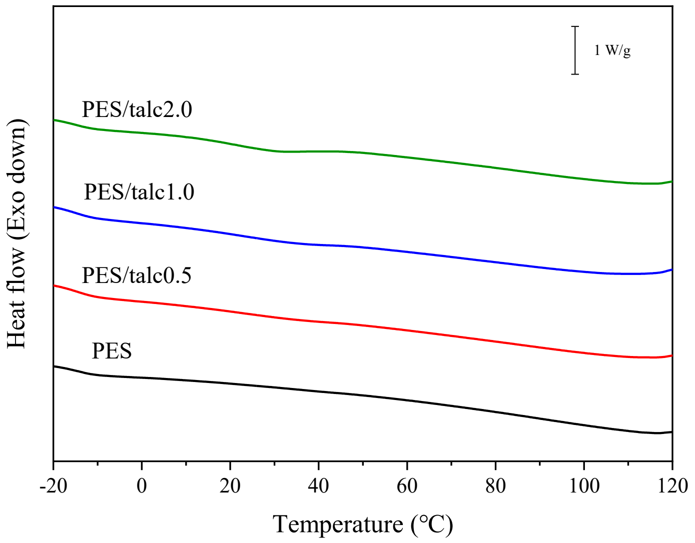

Figure S1. DSC cooling traces of neat PES and PES/talc composites at 60 ºC/min.

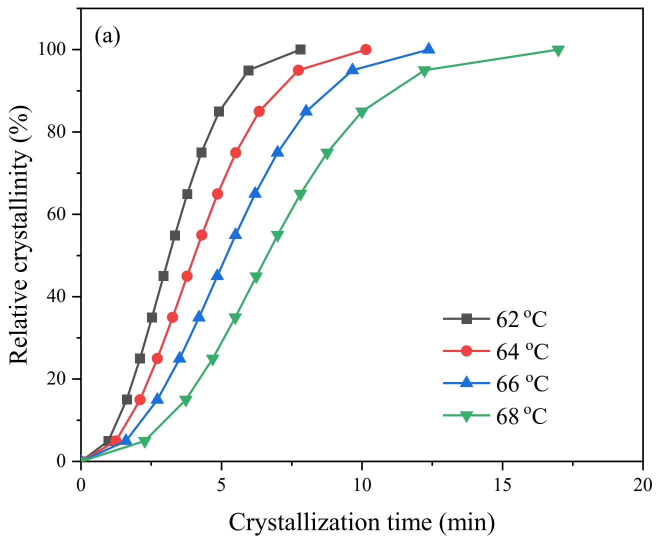

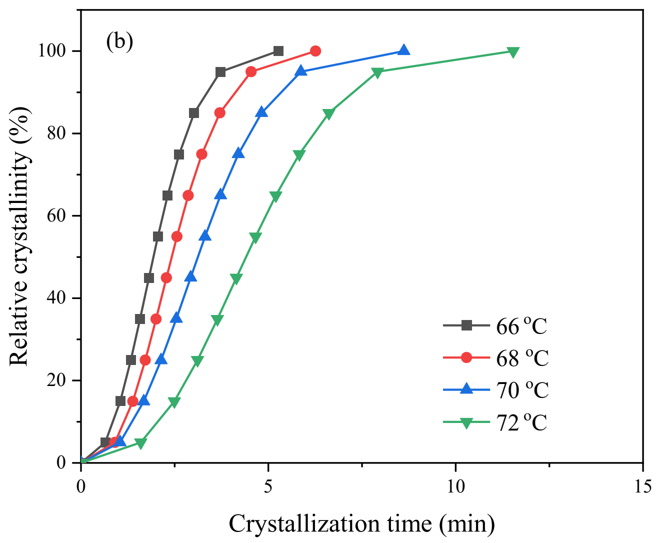

Figure S2. Plots of relative crystallinity versus crystallization time for (a) PES/talc0.5 and (b) PES/talc2.0.

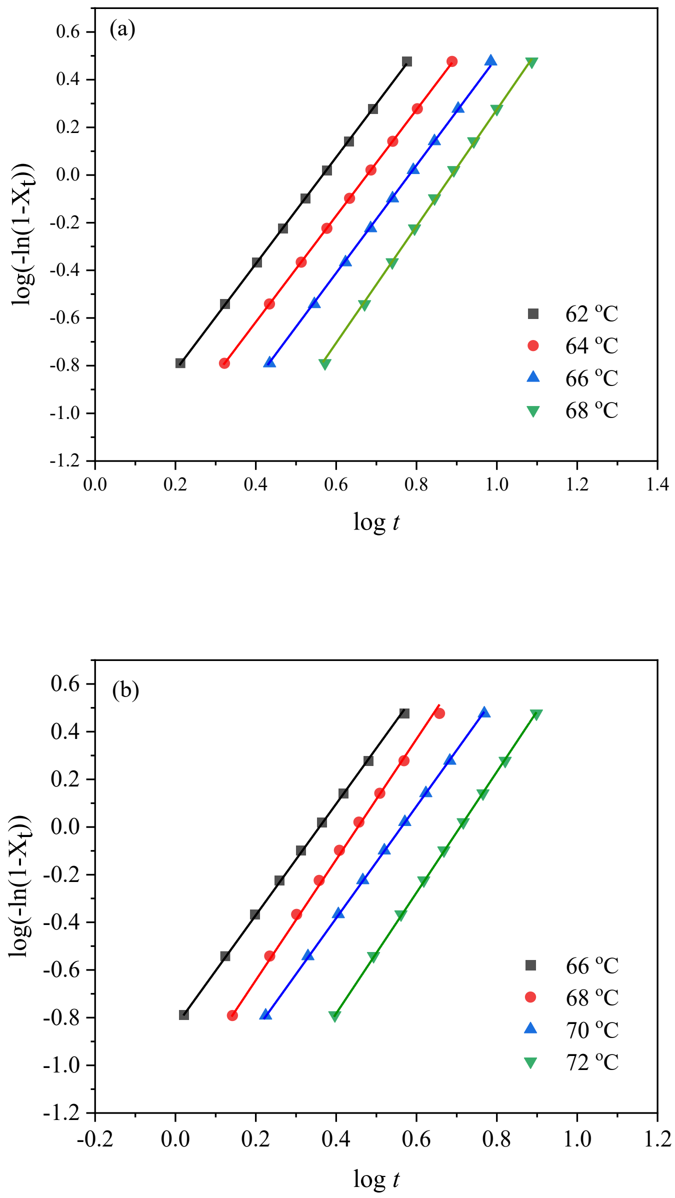

Figure S3. Avrami plots of (a) PES/talc0.5 and (b) PES/talc2.0.
